# Applying the Behavioural and Social Sciences Research (BSSR) Functional Framework to HIV Cure Research

**DOI:** 10.1002/jia2.25404

**Published:** 2019-10-30

**Authors:** Karine Dubé, Judith D Auerbach, Michael J Stirratt, Paul Gaist

**Affiliations:** ^1^ UNC Gillings School of Global Public Health University of North Carolina Chapel Hill NC USA; ^2^ School of Medicine University of California San Francisco San Francisco CA USA; ^3^ Division of AIDS Research (DAR) National Institute of Mental Health National Institutes of Health Bethesda MD USA; ^4^ Office of AIDS Research Division of Program Coordination, Planning, and Strategic Initiatives Office of the Director National Institutes of Health Bethesda MD USA

**Keywords:** Behavioural and Social Sciences Research (BSSR), functional framework, HIV cure research, HIV remission, analytical treatment interruption, people living with HIV

## Abstract

**Introduction:**

The search for an HIV cure involves important behavioural and social processes that complement the domains of biomedicine. However, the field has yet to tap into the full potential of behavioural and social sciences research (BSSR). In this article, we apply Gaist and Stirratt’s BSSR Functional Framework to the field of HIV cure research.

**Discussion:**

The BSSR Functional Framework describes four key research domains: (1) basic BSSR (understanding basic behavioural and social factors), (2) elemental BSSR (advancing behavioural and social interventions), (3) supportive BSSR (strengthening biomedically focused clinical trials), and (4) integrative BSSR (building multi‐disciplinary combination approaches for real‐world implementation). In revisiting and applying the BSSR Functional Framework, we clarify the importance of BSSR in HIV cure research by drawing attention to such things as: how language and communication affect the meaning of “cure” to people living with HIV (PLHIV) and broader communities; how cure affects the identity and social position of PLHIV; counselling and support interventions to address the psychosocial needs and concerns of study participants related to analytical treatment interruptions (ATIs); risk reduction in the course of ATI study participation; motivation, acceptability, and decision‐making processes of potential study participants related to different cure strategies; HIV care providers’ perceptions and attitudes about their patients’ participation in cure research; potential social harms or adverse social events associated with cure research participation; and the scalability of a proven cure strategy in the context of further advances in HIV prevention and treatment. We also discuss the BSSR Functional Framework in the context of ATIs, which involve processes at the confluence of the BSSR domains.

**Conclusions:**

To move HIV cure regimens through the translational research pathway, attention will need to be paid to both biomedical and socio‐behavioural elements. BSSR can contribute an improved understanding of the human and social dimensions related to HIV cure research and the eventual application of HIV cure regimens. The BSSR Functional Framework provides a way to identify advances, gaps and opportunities to craft an integrated, multi‐disciplinary approach at all stages of cure research to ensure the real‐world applicability of any strategy that shows promise.

## 1 Introduction

A cure for HIV infection has been a hoped‐for goal since the virus was first identified. Despite tremendous scientific advances in preventing and treating HIV, there still is no effective curative intervention for HIV infection 1. The U.S. Food and Drug Administration (FDA) defines HIV cure research as “any investigation that evaluates: (1) a therapeutic intervention or approach that controls or eliminates HIV infection to the point that no further medication interventions are needed to maintain health, and (2) preliminary scientific concepts that might lead to such a therapeutic intervention” 2. Current research on strategies for an HIV cure therefore includes work on HIV eradication as well as sustained antiretroviral treatment (ART)‐free viral remission. Over 250 HIV cure‐related studies have or are being conducted worldwide 3. Examples of strategies under investigation include latent‐reversing agents, gene therapies, stem cell transplants, early ART, and immune‐based strategies, administered alone or in combination 3, 4. It is well understood that initial HIV cure clinical studies will not lead to complete removal of the virus and will pose substantial risks to study participants 5, 6. Some HIV cure clinical studies will require an intensively monitored ART pause (IMAP), also often referred to as analytical treatment interruption (ATI), to demonstrate efficacy of interventions 7.

Most HIV cure research has remained in the realm of pre‐clinical, clinical and translational sciences 8. Yet the search for an HIV cure involves important behavioural and social processes that complement the domains of biomedicine 8, 9, 10. To move cure regimens through the translational research pathway, attention will need to be paid to both biomedical and socio‐behavioural elements 11. While the behavioural and social sciences have made important contributions to HIV prevention 12, 13, 14, 15, 16 and treatment research 17, the HIV cure research field has yet to tap into the full potential of behavioural and social sciences research (BSSR). We therefore have a unique opportunity to assess and advance the state of BSSR related to HIV cure.

**Figure 1:**
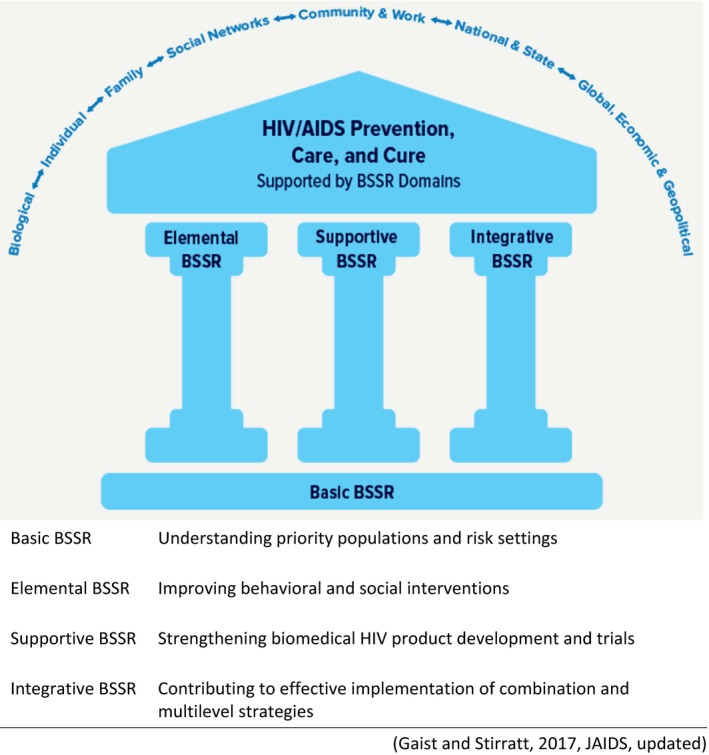
Behavioural and Social Sciences Research (BSSR) Functional Framework and its four domains 17

In this article, we apply Gaist and Stirratt’s BSSR Functional Framework 17 to the HIV cure research field. This framework describes four key domains of BSSR in HIV research, as illustrated in Figure 1: (1) basic BSSR (understanding basic behavioural and social factors), (2) elemental BSSR (advancing behavioural and social interventions), (3) supportive BSSR (strengthening biomedically focused clinical trials), and (4) integrative BSSR (building multi‐disciplinary combination approaches for real‐world implementation). In applying the BSSR Functional Framework, our aims are twofold: (1) to clarify the importance of BSSR in HIV cure research, and (2) to inform possible future research topics and directions. We also briefly discuss the BSSR Functional Framework in the context of ATIs which involve processes at the confluence of the four BSSR domains. The application of the BSSR Functional Framework to HIV cure research, and to the ATI example in particular, serves as a guiding tool with which to envision a rich multi‐disciplinary BSSR agenda related to HIV cure research. A coherent BSSR agenda for HIV cure research and a coordinated approach for the inclusion of BSSR at all stages of translational HIV cure development are needed if we are to most effectively achieve and advance HIV cure efforts.

### 1.1 The Intersection of HIV Cure Research with Behavioural and Social Sciences Research

Biomedical HIV cure studies are highly complex and multi‐faceted. Efforts will be expanded considerably in the coming years, requiring more people to participate in research 10. HIV cure research is conducted against a background of highly effective HIV ART 18. Importantly, early HIV cure research experiments will not be curative and will involve novel interventions with unknown toxicities, with the potential to cause clinical and psychological harms 5, 8. The heterogeneity of HIV cure strategies will require a multi‐disciplinary approach relying on the strengths and methods of multiple intersecting fields 8. BSSR can become a critical adjunct to ongoing biomedical HIV cure research efforts, as it has in the fields of HIV prevention, HIV treatment and oncology 19, by contributing methods to assess community knowledge, to improve behavioural and social approaches to risk reduction—particularly during ATIs—and to ensure a patient‐centred HIV cure research agenda 20. While BSSR refers specifically to a number of scientific disciplines (e.g. psychology, sociology, anthropology, economics, political science, etc.), it often intersects with community engagement and ethics, distinct domains that also are critical to HIV cure research 10, 15, 21.

Following is an articulation of possible BSSR HIV cure research topics and directions organized by the four domains of the BSSR Functional Framework. Given that BSSR related to HIV cure is a nascent field, we illustrate our points with examples from peer‐reviewed papers published in HIV‐related journals in the last five years (2014 to 2019). We recognize that the four BSSR domains are relational and can overlap, reflecting the framework’s ability to highlight research questions relevant to one or more domain. In Table 1, we summarize possible topics and directions that emerge from the application of the BSSR Functional Framework to HIV cure research. Determining how to prioritize these will require ongoing stakeholder engagement in diverse settings.

**Table 1:** Possible topics and directions from the application of the BSSR Functional Framework to HIV cure research

**Basic BSSR: understanding behavioural and social factors**
Defining use of language to define HIV cure research
Understanding community perceptions and knowledge of HIV cure research
Framing expectations around HIV cure research
Examining the construction and management of HIV‐related identities
Assessing the social meaning of finding a cure for HIV infection
Understanding views of becoming detectable/undetectable and how the U = U movement shapes desires to engage in HIV cure research
**Elemental BSSR: Advancing Behavioural and Social Interventions**
Designing counselling and support interventions to address psychological needs related to ATIs
Implementing behavioural risk‐reduction strategies during HIV cure and ATI studies to minimize third‐party risks (e.g., counselling, PrEP provision, adherence to partner protection measures, HIV testing referral)
Developing and implementing HIV stigma reduction interventions
**Supportive BSSR: Strengthening the Design and Outcomes of Biomedically Focused Clinical Trials**
Determining desirable target approach and product profiles for sustained antiretroviral (ART)‐free HIV cure regimens
Examining acceptability of specific HIV cure research strategies
Assessing PLHIV’s (1) willingness to participate in HIV cure research, (2) risk acceptability thresholds for interventions and procedures, (3) barriers and motivators to participation, and (4) acceptability of ATI‐related parameters
Understanding HIV cure researchers’ and HIV care providers’ (1) willingness to refer patients, (2) role of patient‐provider relationships, and (3) shared decision making for cure research participation
Improving informed consent processes and understanding of risks and benefits of HIV cure research
Integrating patient‐reported measures during the course of HIV cure research participation to examine: (1) factors affecting decisions to participate in research (both accepter and decliner assessments), (2) reports of longitudinal participant experiences (with HIV cure research interventions, ATIs, and study procedures), (3) psychosocial aspects of HIV cure research participation, and (4) participant‐centred outcomes
Assessing and supporting adherence to HIV testing and viral load monitoring schedules, as well as ATIs, in cure clinical trial protocols
Integrating strategies to mitigate social impacts and harms during trial participation
Understanding factors affecting or enhancing the engagement and involvement of diverse and under‐represented populations in research, such as women and minority groups
**Integrative BSSR: Advancing Implementation of Integrated, Combination, and Multi‐Disciplinary Approaches**
Developing decision tools to help people living with HIV make informed decisions and choices about any available treatment and cure strategies
Testing behavioural interventions to support patient retention and completion of future HIV cure regimens
Anticipating research needs on factors affecting future real‐world implementation of HIV cure research strategies, including, but not limited to: (1) infrastructure, staffing and training requirements, (2) monitoring of drug resistance and viral loads, (3) co‐morbidities and poly‐pharmacy, and (4) intervening factors such as injecting drug use, mental health issues, intimate partner violence, resilience and food security
Developing HIV cure strategies with scalability considerations
Integrating cost‐effectiveness research and anticipating performance benchmarks for real‐world implementation

## 2 Discussion

### 2.1 BASIC BSSR: understanding behavioural and social factors

Basic BSSR improves understanding of the individual, interpersonal, community and structural factors that are relevant to HIV cure research 17. Basic BSSR can provide the empirical foundation to understand values, beliefs, perceptions and lived experiences of key populations and communities of interest that will be vital to planning successful HIV cure clinical trial conduct, and eventual cure rollout 17.

An important aspect of conducting HIV cure research will involve communicating and framing expectations around early‐phase HIV cure experiments and scientific advances 5. Stakeholders often overestimate the possibility of clinical benefits and underestimate the likelihood of risks in early‐phase clinical research in general 22, 23, and the same is true in early‐phase HIV cure research 6, 24, 25, 26. While benefits of HIV cure research at this stage should be evaluated in terms of the production of incremental scientific knowledge 5, an increasing number of social sciences studies reveal that study participants in HIV cure research strongly value the benefits of inclusion (such as increased HIV knowledge or psychosocial benefits) 25, 27. Cases of sudden viral relapse following long‐term HIV cure remind us of the need to carefully calibrate expectations around expected clinical benefits in the community 28, 29. For example, the cases of the so‐called “Berlin” and “London” patients offer great encouragement, yet reports also may have raised hopes that a cure or remission for HIV is imminent without clarifying how and why the haematopoietic stem cell transplantation employed in these exceptional cases does not represent a viable wide‐scale strategy worldwide 30, 31.

To assist communications about HIV cure research, Basic BSSR is needed on how communities understand HIV cure, as well as the biomedical science behind it, and on how basic community literacy can support translation of HIV cure research information 8. A survey of people living with HIV (PLHIV) in the U.S. showed that respondents held various notions of cure or remission, including having HIV completely eliminated from the body, not being able to transmit HIV to others, not requiring HIV treatment now or in the future, testing negative on the HIV antibody test or reversing immune system damage 32. Chu and colleagues interviewed people who inject drugs in China about the social meaning of curing HIV infection, and found value in combining both “the science of treating disease with the art of healing illness” in assessing community needs 33. Similarly, Newman and colleagues described “mental models” around biomedical HIV interventions that can go a long way in informing the context of clinical trial implementation 34, 35. Notably, a public understanding of science (PUS) approach can reveal how the public uses different sources of knowledge, and integrates it in decision‐making 8, 36.

There is a growing literature examining the role of language in HIV cure research 5. The HIV cure research terminology has most often been used in narrow biomedical contexts, in isolation of the social and psychological contexts of HIV infection 37, 38. The label “cure” may overestimate clinical benefits, and its use has been discouraged in informed consent forms 24. Scholars critically reviewed three frameworks used to describe HIV cure research: “sterilizing versus functional” cure, sustained virological response and clinical remission (borrowed from oncology) 37, favouring “remission” because it underscored the need for vigilance around the possibility of relapse 37. Newton *et al*. revisited the “sterilizing cure” terminology, calling for more patient‐centred perspectives as “sterilization” was associated with concepts of disinfection, cleansing and coerced sterilization 39. Sylla and colleagues found that PLHIV considered a cure to be the complete elimination of all HIV from the body 40. The National Institutes of Health (NIH) uses the terms “viral eradication” and “sustained ART‐free HIV remission” as well as “research towards an HIV cure” 41, 42. Julg *et al*. suggested other possible alternatives to describe HIV cure science, such as “drug‐free viral control” and “durable viral load suppression” 43.

Basic BSSR should further address concepts of personal identity in relationship to HIV cure. While living with HIV has been stigmatized in multiple contexts, it has been valorized in others 44. For some, a cure for HIV may challenge a deeply entrenched identity—that of being HIV positive 8. Being positive has gained powerful currency in the HIV response and has been codified in the 1983 Denver Principles and Greater Involvement of People Living with HIV/AIDS (GIPA), and institutionalized in advocacy networks of people with HIV. PLHIV have been encouraged to live positively and to “come out” 8 and advocate for themselves and their peers, reflecting a form of “biological citizenship” 45. Early BSSR studies indicate that many PLHIV have adjusted well to the chronic condition of HIV and life‐long ART adherence, and have learned to “co‐exist” with the disease 40, 46, 47. In some cases, living with HIV is associated with receiving social protection, such as social security, disability or housing benefits that may not be available to others 48, 49. More BSSR is needed to understand how HIV cure research would affect the fluidity of HIV‐related identities, memberships and social positions.

Achieving durable viral suppression, as operationalized by having a clinically undetectable viral load, has become a major milestone in the therapeutic trajectory of PLHIV 50. Not only does it improve one’s health, but it also prevents sexual transmission of HIV. The science of “treatment as prevention” (TasP) has been translated into a hugely successful communications campaign of “Undetectable = Untransmittable” or “U = U.” It is important to examine how communities understand viral suppression and “U = U” in relation to HIV cure research (and cure itself), and how this affects their priorities. For example, there can be an inherent tension in cure studies utilizing ATIs that precipitate rebounds in viraemia and the U = U concept for sexual transmission of HIV 51. We make a distinction between 1) durable viral suppression from using ART as prescribed and having longstanding clinically undetectable viral load test results versus 2) sustained ART‐free HIV remission meaning longstanding undetectable circulating replication‐competent HIV by research‐grade testing without continued use of ART.

### 2.2 Elemental BSSR: advancing behavioural and social interventions

Elemental BSSR encompasses research that takes understandings from basic research on behavioural and social conditions affecting HIV prevention, treatment, care, and cure outcomes and develops evidence‐based interventions (i.e. tools, strategies and practices) to address them. It includes risk reduction interventions that address sexual and drug using behaviours, as well as efforts to ameliorate HIV and intersectional stigma (defined as the crossing of HIV stigma with stigma/prejudice towards key populations) 17. HIV cure research may benefit from Elemental BSSR interventions in at least three ways.

First, HIV cure research will require counselling and support interventions to address psychosocial needs and concerns related to ATIs. Psychosocial and mental aspects of HIV cure research should not be underestimated, particularly as the field moves towards open‐ended ATI protocol designs 43. Psychosocial assessments and support could be helpful across the entire ATI trajectory, including the experience of waiting for viral rebound, the return of viraemia, staying viraemic for extended periods until viral set point is achieved, and returning to a virally suppressed state. Moreover special attention should be given to those who achieve post‐treatment control, or experience intercurrent events including acute retroviral syndrome, emergent drug resistance mutations or secondary HIV transmission events. PLHIV may feel a deep tension between the altruistic desire to advance HIV cure science and the maximization of their own safety and protection of sexual partners. Biomedical HIV cure research teams should ensure adequate mental health analysis and support throughout the entire course of research participation. Existing psychosocial counselling interventions developed through Elemental BSSR can be leveraged as a starting point for this 52.

Second, Elemental BSSR has provided a set of evidence‐based behavioural risk reduction interventions which will be valuable to HIV cure studies 17. Attention should be paid to sexual risk reduction during the course of ATIs, because HIV RNA can rebound rapidly in blood and semen in the absence of ART 53. Secondary HIV transmission events have occurred in the context of therapeutic vaccine trials 53, 54. This highlights the importance of providing HIV cure trial participants with effective counselling and skill building for behavioural risk reduction while undergoing ATIs. Relatedly, biomedical HIV cure research teams will need to provide adequate counselling on safer sex practices, HIV testing and PrEP to partners without HIV of those undergoing ATIs 43, 55, 56.

Third, HIV stigma reduction interventions could broadly facilitate entry into cure research. HIV‐related stigma remains one of the foremost challenges hindering efforts to tackle the epidemic 17. In the HIV cure research context, stigma can be both a barrier and motivator to advancing biomedical science 57. As a barrier, there are risks associated with individuals discovering that a person is participating in research. The frequency of study visits for viral load monitoring increases this risk. As a motivator, there is the desire to eliminate HIV‐associated stigma by eliminating HIV. This may enhance altruistic participation in research, including the agreement to take on risks or undergo physical pain. More research is needed to understand how ATIs and unpredictable rebounds in viremia affect internalized and externalized stigma and self‐image. Focus groups showed that PLHIV in the U.S. valued the possible de‐stigmatizing effect of no longer living with HIV, and the sense of freedom and liberation that would come with completely eliminating HIV 40. Yet, similar research in China indicated that intersectional stigma was so pervasive in specific groups, such as men who have sex with men or injecting drug users, that a cure would have limited effects on stigma 46, 58, 59. Stigma remains a multi‐dimensional issue deeply rooted in social, behavioural and cultural determinants requiring further investigation and intervention in the HIV cure research context.

### 2.3 Supportive BSSR: strengthening the design and outcomes of biomedically focused clinical trials

Supportive BSSR strengthens the design and conduct of biomedically oriented HIV clinical trials, in all phases of research. Corneli and colleagues presented a typology of approaches used to complement HIV prevention clinical trials, many of which also are relevant to HIV cure research 14. This includes formative research to inform planning of the trial design and to determine whether the investigational product is acceptable to communities and meets their needs 14.

People living with HIV can provide valuable information to advance the development of HIV cure research strategies by assessing the feasibility and acceptability of target approaches and identifying preferred regimen characteristics 17. Desirable attributes of hypothetical approaches can be evaluated through quantitative, qualitative or mixed methods, and through techniques such as conjoint analyses 17. Such assessments can help prioritize strategies currently under development and refine approaches and regimens to increase their acceptability 60. Most HIV Cure‐related BSSR has been conducted in high‐income settings, whereas the utility of novel HIV therapies, including an HIV cure, may be greatest in low‐income contexts, where barriers persist around ART access and daily adherence.

There have been a number of studies assessing willingness to participate in HIV cure research, risk acceptability, barriers and motivators to participation, as well as perceptions, beliefs, needs and concerns of potential study participants around the world 26, 40, 48, 51, 61, 62, 63, 64, 65, 66. Early findings indicate that there likely will be disagreements between stakeholders about what constitutes acceptable risks for HIV cure studies, and a triangulation of these views will be necessary 48, 65. Furthermore, the margin of risk that ought to be tolerated by withdrawing ART even temporarily and under rigorous monitoring is smaller than in the early days of the HIV epidemic when no life‐saving option was available 63. Preliminary supportive BSSR revealed a complex matrix of demographic, experiential, psychosocial, ethical, logistical, economic and evidence‐based factors influencing decisions to participate in HIV cure research 48, 67. Supportive BSSR could also help understand how insights from PLHIV, and relevant stakeholders, are collectively constructed, and how these could in turn strengthen the design and outcomes of biomedically focused clinical trials.

While progress has been made to better understand factors that motivate or deter participation in HIV cure research in general, much remains to be known about factors affecting participation in *specific* HIV cure research strategies or protocols. For example, focus groups conducted in the northwestern region of the United States revealed that none of the participants living with HIV had previously heard of cell and gene therapy as an investigational HIV cure strategy, and once hearing about it, respondents reported aversion for any investigation that would interfere with genes 68. It is highly possible that an effective cure regimen will require a combination of approaches to maintain durable ART‐free HIV suppression, analogous to combination antiretroviral therapy 69, and more research is needed to understand acceptability of combination HIV cure research strategies.

Although Supportive BSSR has focused on the perceptions of PLHIV and biomedical researchers, less inquiry has been directed towards the perceptions of HIV care providers, particularly those not involved in HIV cure research. A key lesson from the cancer field is that decisions to participate in research depend strongly on the existence of a trusting relationship with care providers 70. The rise of the “expert patient” in HIV cure requires an increased emphasis on value‐based frameworks and shared decision‐making for assessing the merits of various therapeutic and research options 45, 71, 72. To become eligible for participation in HIV cure research protocols, PLHIV may need to switch drug regimens to prevent emerging drug resistance while off ART. For example, non‐nucleoside reverse transcriptase inhibitors (NNRTIs)‐based regimens should be replaced with protease inhibitors or integrase inhibitors before ATIs to reduce likelihood of developing resistant virus. If drug resistance occurs during ATIs, participants also must switch regimens. More research is needed on how experiences with HIV treatment, and the possibility of switching regimens, affect desires to engage in HIV cure research.

In general, ATIs remains one of the most controversial aspects of conducting HIV cure research 73. BSSR methods can help the field come to consensus around critical parameters and safeguards to maximize ATIs' utility while minimizing risks 43, 74, 75, 76. Supportive BSSR can also help in understanding various aspects of informed consent related to HIV cure research, including how participants understand the risks and benefits of research 24, 25, 77. BSSR can provide strategies to measure adherence or non‐adherence to ART and ATIs 78. Another important behavioural factor involved during ATIs will be adhering to viral load testing procedures. Current technologies and protocol designs do not allow participants to perform home‐based biospecimen collection for regular viral load testing. Home‐based viral load testing, which could be accomplished through careful self‐collection and postal mailing of dried blood spots 79, would be more convenient and reduce the burden of participation, but would require confirmatory testing at clinical research sites. In any case, due to the need for regular and frequent viral load testing, HIV cure research involving ATIs likely will occur only in settings where such technology is available, which may affect HIV cure (research) access. BSSR can elucidate whether and how inequalities emerge and who has such access and who does not.

Supportive BSSR also includes understanding how participants make decisions to participate in HIV cure research. With the support of biomedical HIV cure research teams, socio‐behavioural scientists are increasingly able to understand decision‐making processes of those who decide to participate (or not) in HIV cure research, rather than relying on hypothetical intentions and perceptions 77, 80. Supportive BSSR may also include patient‐reported outcomes (PROs) and patient‐reported experience measures (PREMs) completed by participants to assess the effects of study participation on various critical domains, such as well‐being, symptoms, functioning, adverse events and experiences with interventions, studies and ATIs 18, 81. There is a strong precedent for including PROs/PREMs in oncology research testing similar novel innovations, such as immunotherapies, targeted therapies, small molecules and stem cell transplants 82. Very little data exist about participants’ experiences and quality of life outcomes within HIV cure trials, and their views of these trials after participating 11. Such assessments could inform a regulatory pathway towards an HIV cure 81.

An important legacy of integrative BSSR from HIV prevention is assessing, monitoring and mitigating social harms or adverse social events associated with research participation, such as difficulties with personal relationships (especially sex partners), employment, education, health care, housing, health insurance, disability/life insurance or travel/immigration 83. Social harm reports have become standard procedures in HIV vaccine trials 83, yet have been underutilized in HIV cure research.

Finally, Supportive BSSR can help engage, recruit and enroll populations traditionally underrepresented in HIV cure research, such as women, transgender persons, and members of racial and ethnic minority groups. BSSR approaches can help with the development of materials to support understanding of HIV cure research so that diverse groups can more easily participate in and contribute to HIV cure research. BSSR can help ensure that participants mirror the HIV epidemic when testing HIV cure interventions, so that results are more generalizable, and interventions are safe and effective in all populations 84, 85, 86, 87.

### 2.4 Integrative BSSR: advancing implementation of integrated, combination and multi‐disciplinary approaches

Whereas Supportive BSSR focuses on strengthening biomedical research and clinical trials, Integrative BSSR aims at strengthening real‐world implementation for proven interventions 17. This domain recognizes that biomedical HIV prevention, treatment and cure strategies are fundamentally both biomedical and socio‐behavioural in nature 17. Their successful real‐world implementation therefore requires multi‐disciplinary perspectives and combination and multi‐level strategies to optimize use and impact 17. Integrative BSSR consequently invites consideration of the tools that would help effectuate any proven cure strategy in the future, and how these integrated cure strategies would synergize with HIV prevention, treatment and care.

Any future proven cure would likely emerge within an evolved HIV therapeutic landscape, with the advent of long‐acting ART formulations and emerging treatment modalities such as therapeutic implants. Long‐acting and sustained‐release ART regimens would offer intermittent treatment for PLHIV, and these may blur the boundary of what it means to be in “remission” 88. In a 2018 U.S. survey, 42% (n = 226) of PLHIV said that they would prefer a version of their HIV medications taken at 6‐month intervals via injectables or implants, compared to other HIV treatment or cure regimen options 89. Looking ahead, patient‐centred HIV cure research will require a deep understanding of patient‐participant preferences towards advancements in HIV therapeutics 18. The HIV cure research field will benefit from the development of decision tools designed to help PLHIV make informed choices about available treatment and cure options 90.

Existence of a proven HIV cure may synergize with HIV care engagement efforts, providing further motivation and momentum to overcome barriers to HIV testing, treatment, care and viral suppression. This is particularly relevant as most HIV cure clinical research protocols require PLHIV with viral suppression and no recent viral blips at entry. It is possible that implementation of cure interventions (or interventions inducing durable ART‐free suppression) in routine clinical and public health practice would bring similar requirements.

At the same time, any proven HIV cure strategy likely will require PLHIV to sustain engagement in an extended course of medical care and supervision which could be accompanied by risks and unpleasant side effects. These circumstances conceivably could challenge retention and completion rates for intensive HIV cure regimens, despite high motivation among many PLHIV to achieve a state of HIV cure. HIV care retention rates are suboptimal around the world due to many challenges with the requirements of life‐long engagement 91. This raises questions about whether behavioural interventions will be needed to support patient retention and completion of future HIV cure regimens, even if they only require a fixed duration of time.

Moreover, scalability of Interventions will be a critical Integrative BSSR topic that will determine the societal value of HIV cure regimens 10, 92, 93. Better knowledge about cost‐effectiveness and performance benchmarks (i.e. efficacy, toxicity, relapse rate, durability) will enhance the likelihood that HIV cure regimens can become viable options in the real‐world 94, 95. This will include planning for infrastructure, capacity building, staffing, financing and health systems that should be in place prior to the deployment of interventions 10, 96. There will be issues related to HIV cure equity and access, as we learned from the prior deployment of Hepatitis C cure 10. The high costs initially associated with Hepatitis C could help inform cost considerations for the deployment of an HIV cure regimen. As with Hepatitis C, the field of HIV cure research will need to grapple with the possibility of reinfection after successful cure. Concurrent developments in the field of long‐acting HIV treatment, Hepatitis B cure research and oncology could also inform considerations for the HIV cure field. Barriers, such as monitoring viral load and HIV drug resistance in settings where the technology and infrastructure may be limited, will need to be overcome to implement an HIV cure in low‐ and middle‐income countries (LMICs) 97. Additionally, anyone fully cured of HIV should be counselled about HIV risk reduction and provided access to PrEP to avoid re‐acquisition of the virus 49.

### 2.5 Addressing issues at the confluence of BSSR domains: the ATI example

The confluence of the Four BSSR domains provides comprehensive, multifaceted exploration for overarching issues in HIV cure research. Using the example of ATIs: (1) Basic BSSR can elucidate the language and communications strategies that will foster understandings of HIV cure research and the rationale for implementing interruptions of HIV treatment in various settings; (2) Elemental BSSR will provide supportive counselling and sexual risk reduction interventions to help clinical trial participants cope with being off ART for extended periods of time; (3) Supportive BSSR will advance the quality of informed consent processes and provide innovative approaches to understand participants’ motivations for joining studies and their experiences during ATIs; and (4) Integrative BSSR will advance understanding towards real‐world implementation of HIV cure.

## 3 Conclusions

BSSR can contribute an improved understanding of the human and social dimensions related to HIV cure research and its eventual application. The BSSR Functional Framework provides a way to identify possible topics, directions, advances, gaps and opportunities in this arena, including how best to craft an integrated, multi‐disciplinary approach at all stages of cure research to ensure the real‐world applicability of any strategy that shows promise. Such an approach requires the integration of biomedical, behavioural, and social sciences research, effective community engagement, and meaningful collaborations among PLHIV, study participants, HIV care providers, researchers, and bioethicists who are willing to transcend disciplinary boundaries and integrate complementary expertise to optimize the search for an HIV cure for all.

## Competing interest

We declare no competing interest.

## Authors’ Contributions

K.D. drafted the initial version of the manuscript. J.D.A., M.J.S. and P.G. reviewed and revised the manuscript. All authors approved the final version of the manuscript.
